# Targeting Folate Metabolism Is Selectively Cytotoxic to Glioma Stem Cells and Effectively Cooperates with Differentiation Therapy to Eliminate Tumor-Initiating Cells in Glioma Xenografts

**DOI:** 10.3390/ijms222111633

**Published:** 2021-10-27

**Authors:** Masashi Okada, Shuhei Suzuki, Keita Togashi, Asuka Sugai, Masahiro Yamamoto, Chifumi Kitanaka

**Affiliations:** 1 Department of Molecular Cancer Science, School of Medicine, Yamagata University, 2-2-2 Iida-Nishi, Yamagata 990-9585, Japan; s-suzuki@med.id.yamagata-u.ac.jp (S.S.); ke-togashi@med.id.yamagata-u.ac.jp (K.T.); s-asuka@med.id.yamagata-u.ac.jp (A.S.); masahiro@med.id.yamagata-u.ac.jp (M.Y.); 2 Department of Clinical Oncology, School of Medicine, Yamagata University, 2-2-2 Iida-Nishi, Yamagata 990-9585, Japan; 3 Department of Ophthalmology and Visual Sciences, School of Medicine, Yamagata University, 2-2-2 Iida-Nishi, Yamagata 990-9585, Japan; 4 Research Institute for Promotion of Medical Sciences, Faculty of Medicine, Yamagata University, Yamagata 990-9585, Japan

**Keywords:** glioma stem cell, anti-folate, RFC-1, brain tumor initiating cells, JNK, serial transplantation assay

## Abstract

Glioblastoma (GBM) is one of the deadliest of all human cancers. Developing therapies targeting GBM cancer stem cells or glioma stem cells (GSCs), which are deemed responsible for the malignancy of GBM due to their therapy resistance and tumor-initiating capacity, is considered key to improving the dismal prognosis of GBM patients. In this study, we found that folate antagonists, such as methotrexate (MTX) and pemetrexed, are selectively cytotoxic to GSCs, but not to their differentiated counterparts, normal fibroblasts, or neural stem cells in vitro, and that the high sensitivity of GCSs to anti-folates may be due to the increased expression of RFC-1/SLC19A1, the reduced folate carrier that transports MTX into cells, in GSCs. Of note, in an in vivo serial transplantation model, MTX alone failed to exhibit anti-GSC effects but promoted the anti-GSC effects of CEP1347, an inducer of GSC differentiation. This suggests that folate metabolism, which plays an essential role specifically in GSCs, is a promising target of anti-GSC therapy, and that the combination of cytotoxic and differentiation therapies may be a novel and promising approach to effectively eliminate cancer stem cells.

## 1. Introduction

Cancer stem cells (CSCs), considered the source of cancer tissue, comprise only a small population of tumor cells, but they remain and cause recurrence after treatment is considered successful at a glance because they are both resistant to therapy and have tumor-initiating ability. Accordingly, the development of a treatment method capable of eliminating CSCs in tumors is key to the prevention of recurrence and cure of cancer. Methods eliminating CSCs in the tumor include physical elimination by cell killing (cytocidal/cytotoxic therapy) and functional elimination by induction of differentiation to non-cancer stem cells (non-CSCs), which lose their tumor-initiating ability (differentiation therapy). Focusing mainly on glioma stem cells (GSCs), which are CSCs of glioblastoma, we elucidated the mechanism of the maintenance of the stem cell properties of GSCs and developed treatment methods leading to loss of these properties through the induction of differentiation by targeting this mechanism [1,2,3,4,5]. However, even after differentiation therapies, a certain proportion of GSCs in the tumor remain, necessitating additional therapies to eliminate the possibility of tumor recurrence from the residual GSCs.

Drug repositioning/repurposing is the use of a drug for a disease different from that previously treated. As a drug for which safety information has been acquired is used, it is possible to save time and money necessary for the development of new drugs [6]. Using the drug repositioning/repurposing technique, we discovered a number of inducers of GSC differentiation [2,3,4]. With a goal of reducing residual GSCs after differentiation therapies, we searched for GSC-killing drugs using a similar approach and identified a number of drugs that selectively inhibit the survival of GSCs, among which were inhibitors of oxidative phosphorylation, which is more strongly activated in GSCs than in non-GSCs [7,8]. Of note, among these GSC-selective cytotoxic drugs were folate antagonists. In this study, we aimed to clarify the mechanism of the selective cytotoxic effects of folate antagonists in vitro and evaluate their GSC-inhibitory effects in vivo.

## 2. Results

### 2.1. MTX Exhibits GSC-Selective Cytotoxic Effects

**Figure 1:**
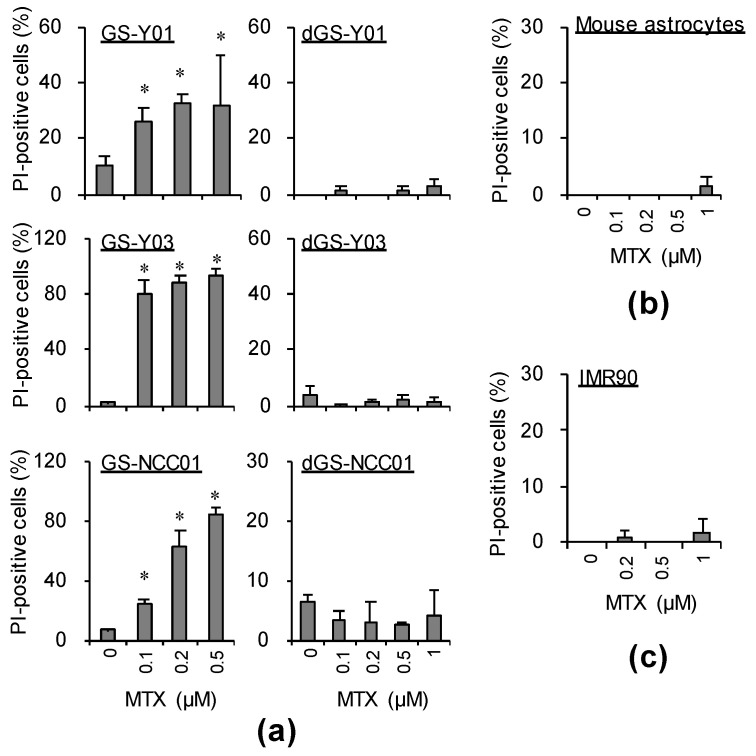
Methotrexate (MTX) induces cell death specifically in glioma stem cells (GSCs), but not in differentiated GSCs. GSCs (GS-Y01, GS-Y03, and GS-NCC01) and differentiated GSCs (dGS-Y01, dGS-Y03, and dGS-NCC01) (**a**), mouse astrocytes (**b**), and IMR90 cells (**c**) treated with the indicated concentrations of methotrexate (MTX) or control solvent treatment for 3 days were analyzed by propidium iodide incorporation assay. Similar results were obtained from two independent biological replicates. Data are presented as means + standard deviation. * *p* < 0.05 vs. control-treated cells by the Mann–Whitney U test.

**Figure 2:**
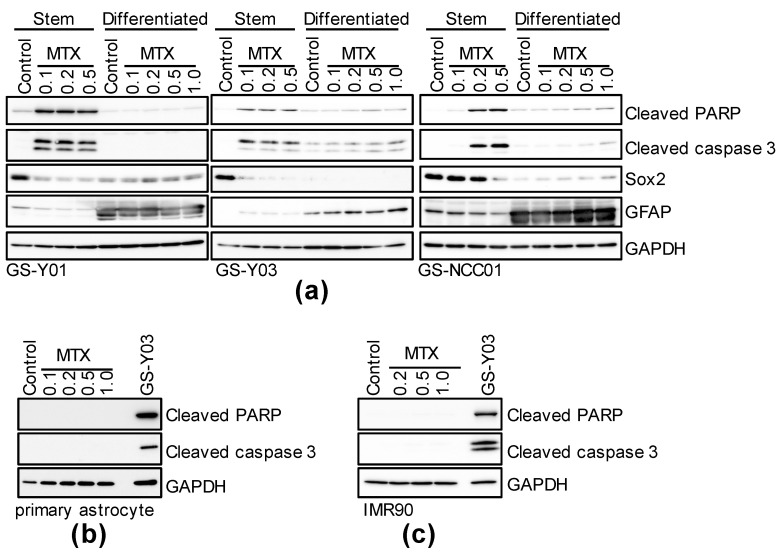
MTX induces caspase-activated cell death in GSCs, but not in differentiated GSCs. GSCs (GS-Y01, GS-Y03, GS-NCC01; Stem or Differentiated, (**a**)), mouse primary astrocytes (**b**), and IMR90 (**c**) treated with the indicated concentrations (μM) of MTX for 3 days or control-treated were analyzed by immunoblotting for the indicated proteins. “GS-Y03” in (**b**,**c**) denotes GS-Y03 cells treated with 0.2 μM MTX for 3 days. Representative images of two independent biological replicates.

We searched for a drug exhibiting GSC-selective cytotoxic effects using GSCs and, as a control, genetically identical (isogenic) differentiated GSCs (differentiated GSCs) acquired through the induction of differentiation of GSCs. As a result, we identified several candidate GSC-selective cytotoxic drugs [7,8], one being methotrexate (MTX). MTX is a folate antagonist currently employed as a therapeutic drug for rheumatism, which also has a long history of use as an antitumor drug to mainly treat malignant lymphoma [9,10,11]. To assess the effects of MTX on GSCs, we investigated whether MTX has cell death-inducing effects on GSCs. First, GSCs (GS-Y01, GS-Y03, and GS-NCC01) were treated with MTX. MTX strongly induced cell death of GSCs in a concentration-dependent manner (Figure 1a, left panels). On the other hand, it exhibited no cell death-inducing effects in isogenic differentiated GSCs which highly expressed the differentiation marker glial fibrillary acidic protein (GFAP) and had low expression of the CSC marker sex-determining region Y-box 2 (SOX2) (dGS-Y01, dGS-Y03, and dGS-NCC01; see Figure 2a), lung normal fibroblasts, IMR90, or primary astrocytes even at a concentration higher than that exhibiting cytotoxicity for GSCs (Figure 1a, right panels, Figure 1b,c). To confirm that these GSC-selective cytotoxic effects were due to the action of MTX as a folate antagonist, we performed a similar investigation using pemetrexed (PEM). PEM is a newly developed folate antagonist that has been approved as a therapeutic drug for non-squamous non-small-cell lung cancer and mesothelioma by the FDA [12,13]. In an experiment similar to that with MTX, GSC-selective cytotoxic effects were noted with PEM (Supplemental Figure S1A,B).

**Figure 3:**
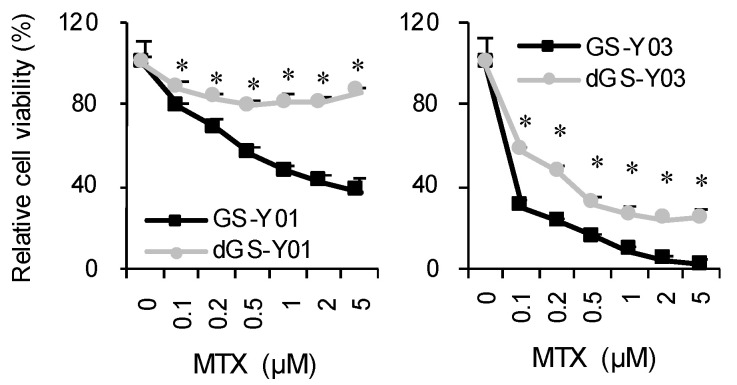
Growth-inhibitory effects of MTX on GSCs and differentiated GSCs. GSCs (GS-Y01, GS-Y03) and differentiated GSCs (dGS-Y01, dGS-Y03) treated with MTX at the indicated concentrations for 3 days were subjected to the WST-8 assay. Their viability relative to control was measured. Values in the graphs represent means + standard deviation from triplicate samples of a representative experiment. Similar results were obtained from two independent biological replicates. * *p* < 0.05 vs. each concentration MTX-treated GSCs by Student’s *t*-test.

Folate antagonists thus exhibited GSC-selective cytotoxic effects. Subsequently, we performed a WST assay to investigate the influence of MTX on overall cell proliferation. MTX markedly inhibited GSC proliferation compared with the proliferation of isogenic differentiated GSCs, consistent with the results of the cell death assay. Of note, the viability of isogenic differentiated GSCs was also reduced in the MTX concentration range confirmed to induce no cell death of isogenic differentiated GSCs (Figure 3), suggesting that MTX inhibits the growth of non-GSCs through a cytostatic, not cytotoxic, mechanism.

### 2.2. Folate Antagonists Induces Apoptotic Cell Death of GSCs

Next, we investigated the mechanism of MTX-induced cell death. First, to investigate the involvement of apoptosis, each GSC subset and its corresponding isogenic differentiated counterpart were treated with MTX, followed by immunoblotting. When these cells were treated with MTX at the same concentration used in Figure 1, expression of apoptotic cell death markers, cleaved PARP and cleaved caspase 3, increased, demonstrating a high correlation with the degree of cell death observed in Figure 1 and Figure 3a. In addition, in primary astrocytes and normal fibroblast IMR90, which were resistant to the induction of cell death by MTX, as shown in Figure 1, no MTX treatment-induced increase in the expression of cleaved PARP or cleaved caspase 3 was noted (Figure 2b,c). Similar results were acquired when PEM was used instead of MTX (Supplemental Figure S1C). This suggests that folate antagonists induce GSC-selective apoptotic cell death.

### 2.3. GSC-Selective Cytotoxic Effects of MTX Are Dependent on RFC-1/SLC19A1

**Figure 4:**
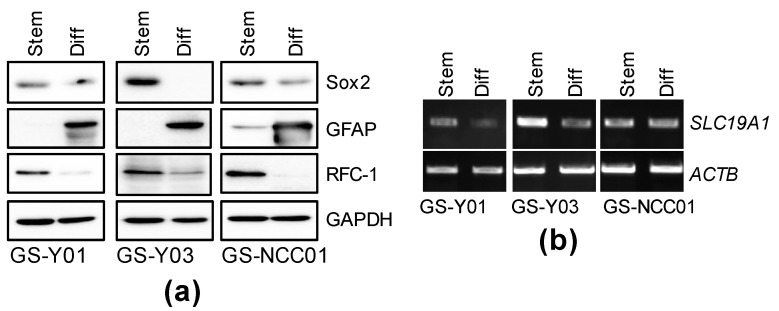
The expression of RFC-1/SLC19A1 in GSCs is reduced by differentiation. GSCs (GS-Y01, GS-Y03, and GS-NCC01; Stem) and differentiated GSCs (Diff) were analyzed by immunoblotting (**a**) or RT-PCR (**b**) for the indicated proteins or mRNAs. Representative images of two biological replicates are shown.

**Figure 5:**
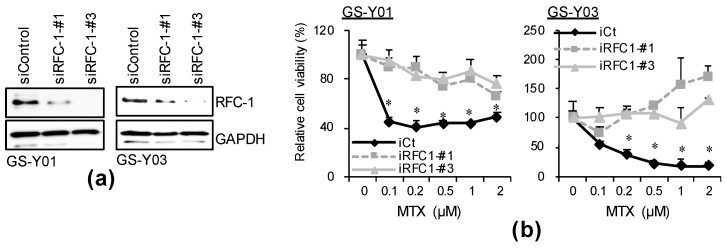
Selective toxicity of MTX to GSCs is dependent on RFC-1 expression. (**a**) GS-Y01 and GS-Y03 cells (2–5 × 10^5^) were transiently transfected with an siRNA against RFC-1 (siRFC-1) or with control siRNA (siControl) and cultured for 4 days. The cells were subjected to immunoblotting analysis for RFC-1 and GAPDH. (**b**) The siRNA-transfected cells were treated with MTX at the indicated concentrations for 3 days and then subjected to WST-8 analysis. * *p* < 0.05 vs. siControl-transfected cells treated with each concentration of MTX by Dunnett’s test.

Folate antagonists acquire pharmacological activity after being incorporated by folate carrier protein and metabolized, and folate receptors (FOLRs), reduced folate carrier (RFC-1/SLC19A1), and proton-coupled folate transporter (PCFT/SLC26A1) are known as folate carrier proteins [14]. Thus, we investigated which folate carrier is expressed in the GSC subsets at the mRNA level. Of the folate carriers, RFC-1/SLC19A1 was strongly expressed in all GSC lines. On the other hand, the expression of FOLR1, 2, and 3 was weak in all GSC subsets, and SLC46A1 was expressed only in GS-Y01 and GS-NCC01 (Supplemental Figure S2). As RFC-1/SLC19A1 was a folate carrier commonly expressed in GSCs at a high level, we investigated whether its expression level is affected by differentiation. The RFC-1 protein expression level decreased after differentiation (Figure 4a), whereas the RFC-1/SLC19A1 mRNA level decreased or was unaffected after differentiation (Figure 4b), suggesting that GSC-selective cytotoxicity of MTX is dependent on the RFC-1 protein expression level. To confirm this hypothesis, an RFC-1/SLC19A1 knockdown experiment was performed. In the control knockdown, cell viability decreased in a MTX concentration-dependent manner. On the other hand, the MTX-induced reduction in cell viability was canceled in the RFC-1/SLC19A1 knockdown (Figure 5). This suggests that in GSCs, the cytotoxic effects of folate antagonists, such as MTX, are exhibited after being incorporated in a manner dependent on the high expression of RFC-1 compared with that in isogenic differentiated GSCs.

### 2.4. MTX Alters the GSC-Inhibitory Effects of Differentiation Induction In Vivo

As strong GSC-selective cytotoxic effects of folate antagonists in vitro were clarified, we investigated the GSC-reducing effects of MTX in vivo. First, to establish the MTX administration conditions, a toxicity test using nude mice was performed referring to previous reports (Supplemental Figure S3) [15,16]. In the early toxicity test, the mice died or markedly lost weight, being intolerable, either under the condition of administration of 50 mg/kg/day MTX for 1 day followed by 6-day consecutive administration of 10 mg/kg/day MTX or under the condition of every 3-day administration of 150 mg/kg/day MTX with leucovorin (LV) rescue. LV is a stable and reduced active form of folate, which is converted to tetrahydrofolate without requiring DHFR, and can bypass the inhibition of tetrahydrofolate synthesis by MTX. We then investigated the following condition: 10-day consecutive administration of MTX at 50 mg/kg/day with LV rescue, and confirmed the absence of clear toxicity (Supplemental Figure S3) [15,17]. Thus, to investigate the possibility that MTX reduces the number of GSCs present in xenograft tumors, a serial transplantation assay was performed using this condition without toxicity. This serial transplantation assay is considered the golden standard of in vivo CSC quantitative methods capable of quantifying the influence of treatment of primary tumors on the number of CSCs existing in the tumor based on the presence of secondary tumors formed after transplantation [18]. Under this administration condition, no influence of MTX on the general health status of the mice was noted throughout the period of drug administration to treat xenografts, as similarly noted in the toxicity experiment. As expected, MTX had no inhibitory effects on enlargement of the primary tumor formed by subcutaneously transplanted GSCs (Supplemental Figure S4A,B). However, MTX treatment had no significant influence on the survival time of mice in the serial transplantation assay (Supplemental Figure S4C). This suggested that MTX administered alone does not significantly reduce the number of GCSs in the xenograft tumor.

**Figure 6:**
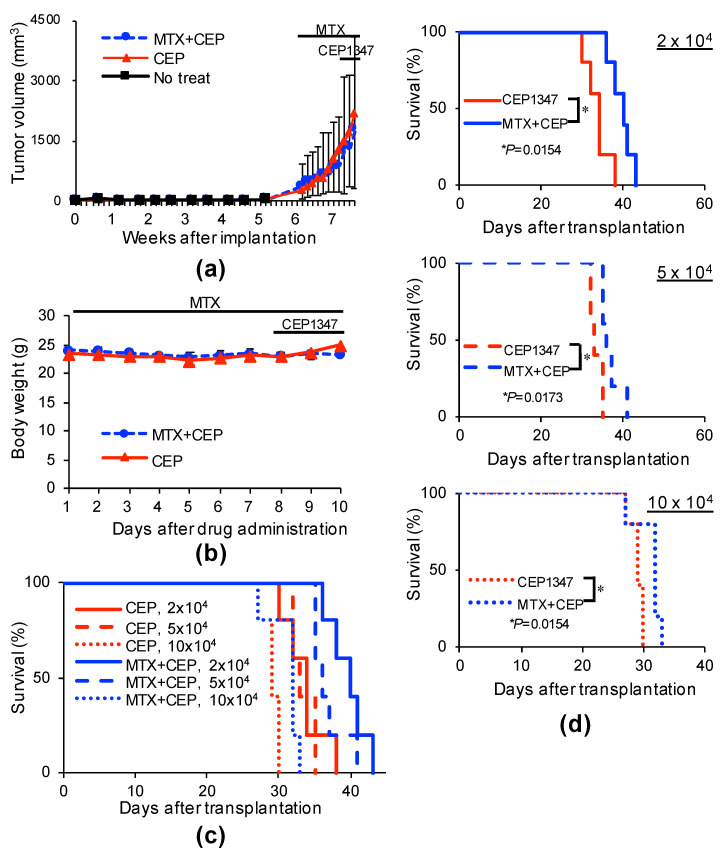
Systemic administration of MTX cooperates with CEP1347 to inhibit tumor-initiating cells in tumors in tumor-bearing mice. (**a**,**b**) Mice implanted subcutaneously with GS-Y03 (1 × 10^6^ cells) were randomized into 2 treatment groups (3 mice per group) 6 weeks after implantation, when the average primary tumor volume reached approximately 300 mm^3^, and received a daily intraperitoneal injection of CEP1347 (CEP; 1.5 mg/kg/day) for 3 consecutive days, as indicated schematically in (**a**,**b**). Mice in the MTX + CEP group received an additional daily intraperitoneal injection of MTX (50 mg/kg/day) followed by intraperitoneal leucovorin (50 mg/kg/day, 4 h after MTX) for 10 consecutive days, which started at the time of randomization and 1 week before the administration of CEP1347. In the combination (MTX + CEP) group, MTX was administered 6 h before CEP1347. One day after the final drug treatment, the subcutaneous tumors were excised and dissociated. Then, the serial dilutions of the dissociated tumor cells were transplanted intracranially into new mice. The volume of the primary tumors treated with CEP1347 or MTX + CEP1347 (MTX + CEP) was assessed at the indicated time points and is presented in the graph as the mean ± SD (**a**), and body weight was monitored at the indicated time points (**b**). Kaplan–Meier survival curves of the mice (*n* = 5 for each group) are shown (**c**). For clarity, the survival curves of mice transplanted with 2 × 10^4^ (upper), 5 × 10^4^ (middle), or 10 × 10^4^ (lower) cells are shown separately in (**d**). * *p* < 0.05 vs. CEP1347-treated mice by the log-rank test.

We next investigated the combined effects of MTX and differentiation induction. To induce differentiation, a JNK pathway inhibitor, CEP1347, discovered as a GSC differentiation inducer by us, was used, and a group treated with CEP1347 alone following the previously reported administration protocol was set as a treatment control group [3,19]. For combination with MTX, a protocol identical to that shown in Supplemental Figure S4 was used, including rescue by LV. In the administration protocol of this combination experiment, there was no notable change, including weight loss, in the general health status of the mice, and enlargement of the primary tumor was not significantly inhibited throughout the drug administration period (Figure 6a,b). These primary tumors were excised on the day following completion of drug administration, tumor cells were re-transplanted to the brain of different nude mice, and the serial transplantation assay was performed. In the animals transplanted with primary tumor-derived tumor cells treated with CEP1347 alone, the survival time decreased as the number of transplanted cells increased, consistent with a previous report [3]. On the other hand, in the CEP1347/MTX combination treatment group, on comparison of the same number of transplanted cells, the survival time was significantly extended compared with that of the CEP1347 monotherapy group (Figure 6c,d). This suggested that GSCs in the primary tumor were further reduced by the combination of CEP1347 with MTX, and implied that although MTX alone is unable to exhibit GSC-inhibitory effects in vivo, it can in combination with a differentiation inducer such as CEP1347.

## 3. Discussion

In this study, we discovered through in vitro experiments that human glioblastoma-derived GSCs are highly sensitive to the folate antagonists MTX and PEM, and that cell death is strongly induced by these drugs. On the other hand, isogenic differentiated GSCs and normal cells, fibroblasts and astrocytes, survived in the presence of folate antagonists. Folate antagonists reduced the cell viability of GSCs to approximately 50% within the concentration range of 0.1–0.2 μM. This concentration range corresponds to the Cmax (0.25–0.6 μM) of MTX administered to rheumatoid arthritis patients [19]. In addition, the blood concentration is 1–10 μM when MTX is administered to treat malignant lymphoma [20]. Comparison of these values suggested that MTX affects GSCs even at a low concentration. PEM is a newly developed folate antagonist capable of inhibiting multiple folate-metabolizing enzymes, and it has been approved as a therapeutic drug for non-squamous non-small-cell lung cancer and mesothelioma by the FDA [12,13]. The Cmax of PEM is 50–200 μg/mL (100–400 μM) [21]. The PEM concentration employed in this study was one-tenth or less of this Cmax, suggesting that PEM is also effective against GSCs at a low concentration. For chemotherapy of glioblastoma developing in the brain, the presence of the blood–brain barrier may be a problem, but high-dose MTX is used in chemotherapy for primary central nervous system lymphoma (PCNSL) developing in the brain because it can pass through the blood–brain barrier via rapid intravenous drip infusion, and the MTX concentration in cerebrospinal fluid reaches 10 μM with this treatment method [22]. The concentration range acquired by this high-dose MTX therapy may not be sufficiently cytotoxic to non-GSCs that comprise the majority of the tumor, but it may be sufficiently cytotoxic to GSCs. In addition, MTX-loaded lipid-core nanocapsules (MTX-LNCs) as another drug delivery system were reported to be effective in experiments using preclinical models [23]. Therefore, not only high-dose MTX therapy, but also MTX-LNCs, may be able to provide sufficient cytotoxicity against GSCs.

However, contrary to the in vitro findings, no decrease in the number of GSCs in the tumor was noted after the administration of MTX alone in the in vivo serial transplantation assay (Supplemental Figure S4). Possible explanations for the absence of a decrease in the number of GSCs in the tumor treated with MTX alone include an insufficient dose of MTX. In addition, as the expression level of dihydrofolate reductase (DHFR), which reduces dihydrofolic acid to tetrahydrofolic acid, reportedly increases in tumor cells immediately after the administration of MTX alone, causing MTX resistance [24], the possibility of involvement of this mechanism was also considered. In support of this possibility, DHFR expression increased in the tumor treated with MTX alone in this experiment (Supplemental Figure S5). Another possibility is that non-GSCs are converted to GSCs immediately after the elimination of GSCs in the tumor by MTX [25], replenishing lost GSCs. Of note, the expression of the stem cell marker SOX2 increased in the tumor treated with MTX alone (Supplemental Figure S5), suggesting that overreacting conversion from non-GSCs to GSCs occurred in response to the exhaustion of GSCs caused by MTX. If the absence of the GSC-inhibitory effects in the tumor treated with MTX alone is due to the conversion of non-GSCs to GSCs, then the combination of a differentiation inducer and a GSC-killing drug may function in such a manner that, whereas the GSC killer reduces residual GSCs after treatment with the differentiation inducer as planned at the beginning of this study, the latter in turn prevents the dedifferentiation process converting non-GSCs to GSCs after treatment with the GSC killer, which may effectively and continuously reduce the number of GSCs in the tumor. Thus, we investigated the combination of MTX with CEP1347, which exhibits differentiation-inducing effects on GSCs [3]. Although no survival time-extending effects by treatment with MTX alone were noted (Supplemental Figure S4), CEP1347 significantly extended the survival time when combined with MTX compared with that in the group administered CEP1347 alone. The survival curve of the CEP1347 monotherapy group (2 × 10^4^) was between the 5 × 10^4^ and 10 × 10^4^ combination groups, suggesting that GSCs in the tumor were reduced to one- ~ two-fifths by the combination with MTX (Figure 6c,d). Although the exact mechanism underlying the strengthened GSC-inhibitory effects by this combination remains to be elucidated, the combination of a CSC differentiation inducer and CSC-killing drug may be a promising CSC-targeting treatment method for glioblastomas and possibly other types of human cancers. The combination with other differentiation inducers previously discovered by us, such as the JNK inhibitors AS602801 and SP600125, and metformin, was not tried in this study, but similar effects of combination therapy with AS602801 may be expected because CEP1347 used in this study is a JNK pathway inhibitor, and AS602801 is also a JNK inhibitor for which a phase I clinical trial (NCT01630252) has been completed and safety information has been acquired [2,26,27].

As folate metabolism is an essential metabolic pathway for DNA synthesis, such as the production of dTMP from dUMP, it has been targeted to treat tumors from the viewpoints of the inhibition of cell proliferation and survival. It was recently reported that one-carbon metabolism, which is a methylation transition pathway involved in amino acid synthesis and the oxidative stress defense mechanism, plays an important role in the maintenance of CSCs. As folate metabolism also plays a role therein, the possibility of metabolizing enzymes as potential targets for GSC-directed therapies has been considered [28,29,30]. In this regard, DHFR was recently reported by Fawal et al. to be necessary for the maintenance of self-renewal capacity in brain tumor-initiating cells (BTIC) [31]. However, this report is different from ours in that we demonstrated that folate metabolism is required for the maintenance of the viability rather than the self-renewal capacity of GSCs and that the in vivo GSC-inhibitory effects of BTIC-targeting treatment were not investigated in the study by Fawal et al. Thus, future clarification of the roles of the numerous molecules involved in folate metabolism in the survival and maintenance of stem cell properties of GSCs should lead to a better understanding of their respective roles in GSCs, and consequently to the development of more effective and safer GSC-targeting treatments. In addition, we discovered that the expression level of reduced folate carrier protein RFC-1/SLC19A1 localized to the cell membrane is higher in GSCs than in isogenic differentiated GSCs. A database search using GlioVis, an expression database utilizing large-scale high-throughput whole genome analysis of human glioma, revealed that the expression level of SLC19A1 was slightly higher in glioblastoma than in non-cancer tissue, but no significant relationship with the outcome was noted (GlioVis; http://gliovis.bioinfo.cnio.es/, accessed on 6 August 2021) [32]. This implies that while the number of CSCs in the tumor increases as the malignancy of glioma progresses, the number of CSCs in the tumor before treatment does not necessarily determine the outcome. Similar to RFC-1, other transporter proteins involved in folate uptake are FOLRs. In the GSCs used in this study, FOLRs were almost undetectable at the mRNA level, exhibited different expression patterns, and had no consistent trend of expression changes upon differentiation at the protein levels (Supplemental Figures S2 and S6). On the other hand, FOLR1 was reported to maintain the stemness of neural stem cells and is involved in their fate in addition to mere survival [33,34]. Although our study suggested that RFC-1 is an essential folate uptake pathway for the GSCs we used in this study, it does not necessarily exclude the need for other FOLRs. There may be GSCs for which other FOLRs are essential. Further studies are necessary. GSC-specific vulnerability based on comparison between GSCs and isogenic differentiated GSCs, as observed in this study, may be a weak point of cancer that is difficult to discover using clinical databases (database employing comparison between cancer tissue and non-cancer tissue), and an approach aiming at discovering this difference (between GSCs and isogenic differentiated GSCs) may be a shortcut to finding a candidate cancer stem cell-targeting therapeutic drug.

In conclusion, this study clarified that the expression level of folate carrier protein RFC-1 is higher in GSCs than in isogenic differentiated GSCs, revealing the high sensitivity of GSCs to folate antagonists. In addition, use of an in vivo model suggested that a folate antagonist, MTX, does not exhibit inhibitory effects by itself, but cooperates with a GSC differentiation inducer to inhibit GSCs in the tumor. This study demonstrated that the combination of a GSC differentiation inducer with a GSC-killing drug is a promising GSC-targeting treatment strategy and may be a milestone in CSC research.

## 4. Materials and Methods

### 4.1. Reagents and Antibodies

Methotrexate, leucovorin (calcium folinate), and CEP1347 (synthesized product code FE29092) were purchased from Tokyo Chemical Industry co., LTD (Tokyo, Japan). Pemetrexed was purchased from FUJIFILM Wako Chemicals (Osaka, Japan). CEP1347 was dissolved in DMSO to prepare a 1 mM stock solution. Methotrexate and pemetrexed were dissolved in 1 M NaOH or DDW to prepare 100 mM and 10 mM stock solutions for in vitro study. Methotrexate and leucovorin were dissolved in 0.1 N NaOH or DDW to prepare 20 mg/mL stock solutions for the mouse study. An antibody against SOX2 (MAB2018) was purchased from R&D Systems Inc. (Minneapolis, MN, USA). Antibodies against cleaved PARP (#9541), cleaved caspase 3 (#9661), GFAP (#3670), and GAPDH (#5174) were purchased from Cell Signaling Technology Inc. (Beverly, MA, USA). Anti-RFC-1 (sc-390948) and anti-FR (sc-515521) were purchased from Santa Cruz Biotechnology, Inc. (Santa Cruz, CA, USA). An antibody against DHFR (NBP1-47727) was purchased from Novus Biologicals, LLC (Centennial, CO, USA).

### 4.2. Cell Culture

The human GSCs used in this study (GS-Y01, GS-Y03, and GS-NCC01) were maintained under the monolayer stem cell culture condition reported previously [1,7,8]. The differentiation of GSCs was induced by culturing cells in DMEM/F-12 medium supplemented with 10% FBS (Thermo Fisher Scientific, Waltham, MA, USA), 100 units/mL of penicillin, and 100 μg/mL of streptomycin for 2 weeks [7,8]. Mouse primary astrocytes were isolated from cerebral cortices of neonatal mice as previously reported [8]. Briefly, collected cerebral cortical tissues were rinsed with chilled phosphate-buffered saline (PBS) and then mechanically dissociated by pipetting. Mouse primary astrocytes were filtered through a 70 μm cell strainer (BD Biosciences, Franklin Lakes, NJ, USA), plated on collagen-I-coated dishes, and maintained in DMEM/F12 medium supplemented with 10% fetal bovine serum, 100 units/mL of penicillin, and 100 μg/mL of streptomycin. The astrocyte cultures were used for experiments on days 7–14 after seeding. Human normal fetal lung fibroblasts, IMR90, were obtained from the American Type Culture Collection (ATCC, Manassas, VA, USA) and maintained in DMEM supplemented with 10% FBS. All IMR90 experiments were performed using cells with a low passage number (<8).

### 4.3. Propidium Iodide Incorporation Assay

To assess cell death, the propidium iodide (PI) incorporation assay was used [8]. In brief, cells were incubated with PI (1 μg/mL) and Hoechst33342 (10 μg/mL) for 5 min at 37 °C. Then, the numbers of PI- and Hoechst-positive cells were scored under a fluorescence microscope (CKX41; Olympus, Tokyo, Japan), and the ratio of PI-positive cells (dead cells) to Hoechst-positive cells (total cells) was calculated.

### 4.4. Cell Viability Assay

Cell viability was determined by the WST-8 assay using Cell Counting Kit-8 (DOJINDO LABORATORIES, Kumamoto, Japan) [8]. Cells (1–5 × 10^3^/well) plated in 96-well collagen I-coated plates were treated with drugs as described in figure legends. Subsequently, WST-8 reagent was added and the cells were incubated for 1–2 h at 37 °C. Absorbance at 450 nm was measured using a microplate reader (iMark; Bio-Rad, Hercules, CA, USA). Relative cell viability was calculated as a percentage of absorbance of treated samples relative to that of controls.

### 4.5. Immunoblotting

Cells were harvested and washed with ice-cold PBS and solubilized in RIPA buffer (10 mM Tris/HCl (pH 7.4), 0.1% sodium dodecyl sulfate (SDS), 0.1% sodium deoxycholate, 1% Nonidet P-40, 150 mM NaCl, 1 mM EDTA, 1.5 mM sodium orthovanadate, 10 mM sodium fluoride, 10 mM sodium pyrophosphate, and protease inhibitor cocktail set III (FUJIFILM Wako Chemicals)). Subsequently, the lysate was immediately mixed with the same volume of 2 × Laemmli buffer (125 mM Tris/HCl (pH 6.8), 4% SDS, 10% glycerol, and 10% 2-mercaptoethanol) and boiled at 95 °C for 10 min. The protein concentration of the cell lysates was measured using a BCA protein assay kit (Pierce Biotechnology, Inc., Rockford, IL, USA). Samples containing equivalent amounts of protein were separated by SDS/polyacrylamide gel electrophoresis and transferred to polyvinylidene difluoride membranes. The membranes were probed with the indicated primary antibodies and appropriate HRP-conjugated secondary antibodies, as recommended by the manufacturer of each antibody. For reprobing of immunoblots, primary and secondary antibodies were stripped from the probed membrane using stripping buffer (2% SDS, 100 mM β-mercaptoethanol, and 62.5 mM Tris-HCl (pH 6.8)). After stripping, the membranes were washed with Tris-buffered saline with Tween 20 and blocked with skim milk. Then, the membranes were reprobed with the appropriate antibodies. Immunoreactive bands were visualized using Immobilon Western Chemiluminescent HRP Substrate (Merck Millipore, Billerica, MA, USA) and detected by a ChemiDoc Touch device (Bio-Rad).

### 4.6. Reverse Transcription-PCR Analysis

Total RNA was extracted from cells using Trizol (Thermo Fisher Scientific) and 1 µg of total RNA was reverse transcribed using the PrimeScript RT reagent kit (Takara Bio Inc., Shiga, Japan) according to the manufacturer’s protocol. Target genes were amplified with Quick Taq HS DyeMix (Toyobo CO., LTD., Osaka, Japan). The sequences of gene-specific primer sets are listed in Supplemental Table S1.

### 4.7. Gene Silencing by siRNA

Synthetic siRNAs against RFC-1 (SLC19A1; HSS185934, HSS185936) and medium GC duplex #2 of Stealth RNAi^TM^ siRNA negative control duplexes were purchased from Thermo Fisher Scientific. Transfection of siRNAs was performed using Lipofectamine RNAiMAX (Thermo Fisher Scientific) according to the manufacturer’s instructions.

### 4.8. Mouse Studies

Mouse xenograft studies were carried out as previously described [1,3]. For subcutaneous implantation, 6–9-week-old male BALB/cAJcl-nu/nu mice (CLEA Japan Inc., Tokyo, Japan) were implanted subcutaneously in the flank region with cells suspended in 200 μL of sterilized PBS under anesthesia of medetomidine, midazolam, and butorphanol (0.3, 4, and 5 mg per kg of body weight, respectively). For serial transplantation, primary tumors treated as described in the legends of Figure 6 and Supplemental Figure S4 were excised, and after being washed in chilled sterile PBS, were transferred into DMEM/F-12, minced with scissors, and incubated in TrypLE^TM^ Express (Thermo Fisher Scientific) for 30 min at 37 °C. The enzymatically dispersed cell mass was filtered through a 70 μm strainer and the dissociated cells were transplanted intracranially into new mice after serial dilution. For systemic administration of MTX, LV, and CEP1347, the stock solution of the drug was diluted in PBS to prepare 200 μL solutions for each injection.

### 4.9. Statistical Analysis

All data are shown as means + standard deviation. Data were analyzed using Student’s *t*-test or the Mann–Whitney U test for comparisons between two groups. For comparisons of more than two groups, data were analyzed using a one-way analysis of variance followed by Dunnett’s test. Mouse survival was evaluated by the Kaplan–Meier method and analyzed using the log-rank test. Differences with a *p*-value < 0.05 were considered significant and are indicated with asterisks in the figures.

## Data Availability

All data is contained the article and there is no repository data.

## Supplementary Materials

The following are available online at https://www.mdpi.com/article/10.3390/ijms222111633/s1.
