# Supplementary material for: Targeting Folate Metabolism Is Selectively Cytotoxic to Glioma Stem Cells and Effectively Cooperates with Differentiation Therapy to Eliminate Tumor-Initiating Cells in Glioma Xenografts
Source: Int J Mol Sci. 2021 Oct 27;22(21):11633. doi: 10.3390/ijms222111633 (PMC8583947; doi:10.3390/ijms222111633)
Supplement: Supplementary file 1 [file ijms-22-11633-s001.zip › ijms-1417194-supplementary.pdf]

Supplementary Materials

Targeting folate metabolism is selectively cytotoxic to glioma stem cells and effectively cooperates with differentiation therapy to eliminate tumor-initiating cells in glioma xenografts.

Masashi Okada[1, *], Shuhei Suzuki[1, 2], Keita Togashi[1, 3], Asuka Sugai[1], Masahiro Yamamoto[1], and Chifumi Kitanaka [1, 4, *]

1 Department of Molecular Cancer Science, Yamagata University School of Medicine, 2-2-2 Iida-nishi, Yamagata 990-9585, Japan; m-okada@med.id.yamagata-u.ac.jp (M.O.); masahiro@med.id.yamagata-u.ac.jp (M.Y.); s-asuka@med.id.yamagata-u.ac.jp (A.S.); ckitanak@med.id.yamagata-u.ac.jp (C.K.)

2 Department of Clinical Oncology, Yamagata University School of Medicine, 2-2-2 Iida-nishi, Yamagata 990-9585, Japan; s-suzuki@med.id.yamagata-u.ac.jp (S.S.)

3 Department of Ophthalmology and Visual Sciences, Yamagata University School of Medicine, 2-2-2 Iida-nishi, Yamagata 990-9585, Japan; ke-togashi@med.id.yamagata-u.ac.jp (K.T.)

4 Research Institute for Promotion of Medical Sciences, Yamagata University Faculty of Medicine, Yamagata, 990-9585 (C.K.)

* Correspondence: m-okada@med.id.yamagata-u.ac.jp (M.O.); and ckitanak@med.id.yamagata-u.ac.jp (C.K.); Tel.: +81-23-628-5214

Supplemental Figure S1

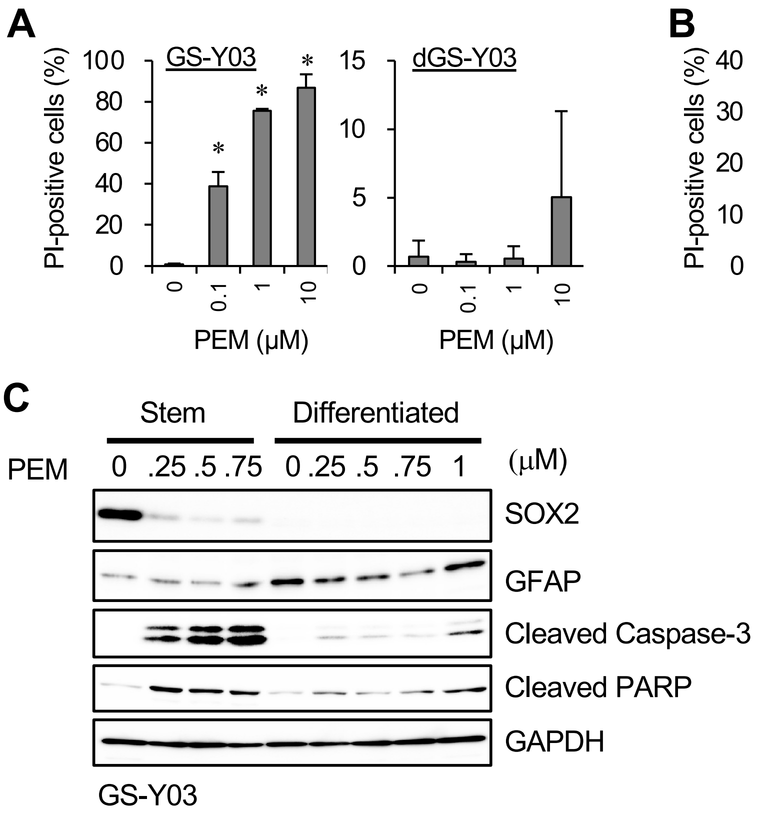

Supplemental Figure S1: Pemetrexed (PEM) induces cell death specifically in glioma stem cells (GSCs) but not in differentiated GSCs. GS-Y03 and differentiated GS-Y03 (dGS-Y03) (A), and IMR90 cells (B) treated with the indicated concentrations of PEM or control solvent treatment for 3 days were analyzed by propidium iodide incorporation assay. Similar results were obtained from two independent biological replicates. Data are presented as means + standard deviation. *$P < 0.05$ vs. control-treated cells by the Mann-Whitney U test. (C) GS-Y03 and dGS-Y03 treated with the indicated concentrations of PEM for 3 days or control-treated were analyzed by immunoblotting for the indicated proteins. Representative images of two independent biological replicates.

Supplemental Figure S2

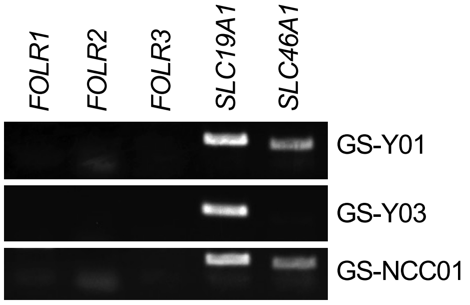

Supplemental Figure S2: The expression of folate carriers in GSCs. GSCs (GS-Y01, GS-Y03, and GS-NCC01) were analyzed by RT-PCR for the indicated mRNAs. Representative images of two biological replicates are shown. Similar results were obtained from two independent biological replicates.

Supplemental Figure S3

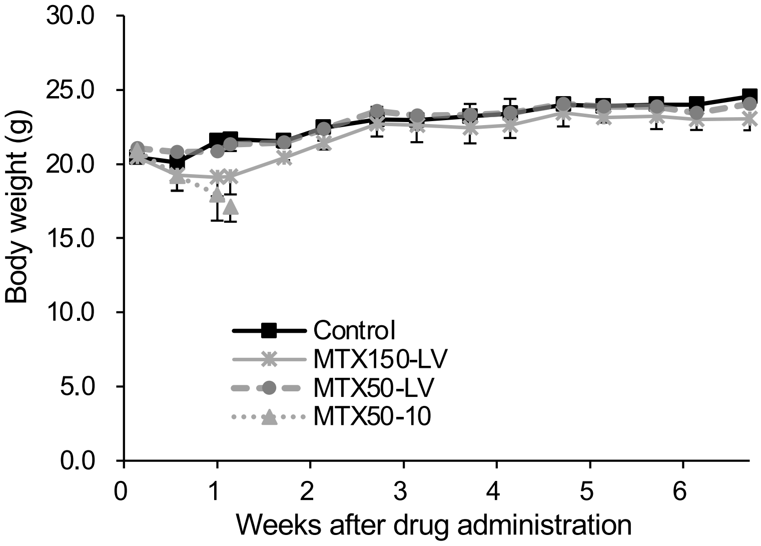

Supplemental Figure S3: The effects of systemic methotrexate (MTX) administration on mouse body weight. Four groups of mice (n=2) were injected intraperitoneally with either vehicle only (Control), 150 mg/kg MTX once every 3 days and 50 mg/kg leucovorin (LV) for 9 consecutive days (MTX150-LV), 50 mg/kg MTX and 50 mg/kg LV for 7 consecutive days (MTX50-LV), or 50 mg/kg MTX once and 10 mg/kg MTX for 6 consecutive days (MTX50-10). LV (50 mg/kg/day) was administered 4 hours after MTX injection. Mouse body weight was measured at the indicated time points. Data are shown as the mean + or - standard deviation for each treatment group.

Supplemental Figure S4

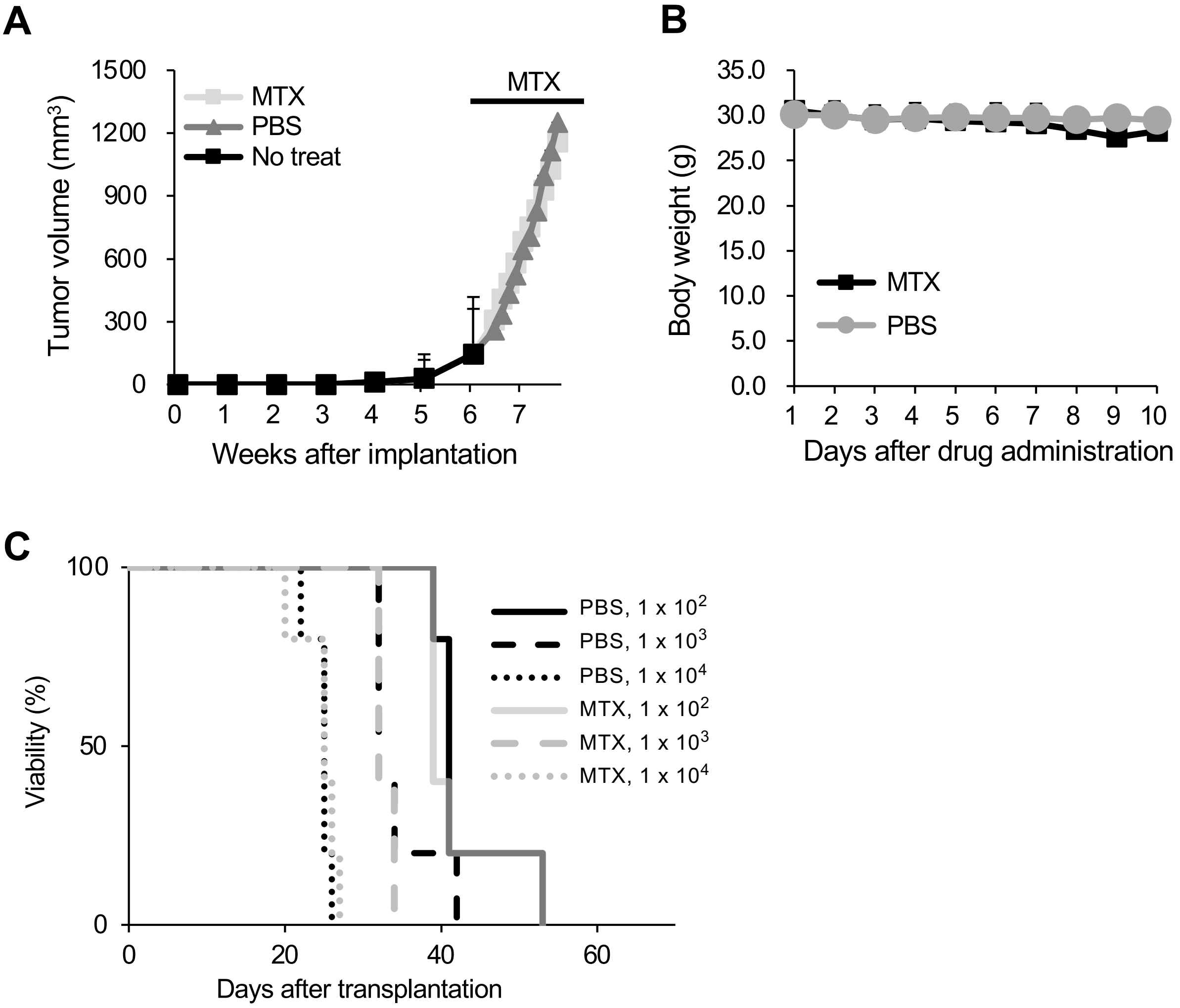

Supplemental Figure S4: Systemic administration of MTX does not inhibit tumor-initiating capacity of GSCs *in vivo*. (A, B) Mice implanted subcutaneously with GS-Y03 (1.5×10^6 cells) were randomized into 2 treatment groups (6 mice per group) 6 weeks after implantation, when the average primary tumor volume reached approximately 150 mm^3, and received a daily intraperitoneal injection of phosphate-buffered saline (PBS, as a control) or MTX (50 mg/kg/day) for 10 consecutive days. Leucovorin (50 mg/kg/day) was administered 4 hours after MTX injection. One day after the final drug treatment, the subcutaneous tumors were excised and dissociated. Then, serial dilutions of the dissociated tumor cells were transplanted intracranially into new mice. Kaplan-Meier survival curves of the mice (n = 5 for each group) are shown (C).

Supplemental Figure S5

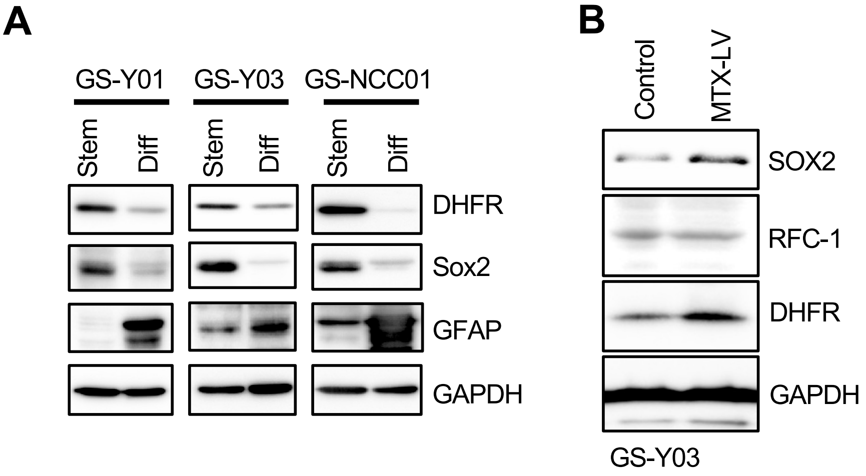

Supplemental Figure S5: The expression of DHFR in GSCs. (A) GSCs (GS-Y01, GS-Y03, and GS-NCC01; stem) and differentiated GSCs (Diff) were analyzed by immunoblotting for the indicated proteins. Representative images of two biological replicates are shown. (B) The indicated proteins were detected by immunoblotting using the cell lysates from dissociated primary tumors shown in Supplemental Figure S4.

Supplemental Figure S6

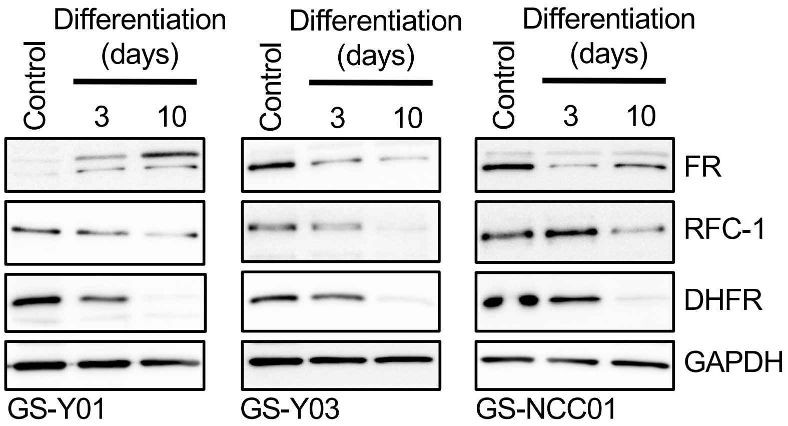

Supplemental Figure S6: The protein expression levels of FR, RFC-1, and DHFR in GSCs. (A) GSCs (GS-Y01, GS-Y03, and GS-NCC01) induced to differentiate for the indicated days were analyzed by immunoblotting for the indicated proteins.

Supplemental Figure S7

Fig3A

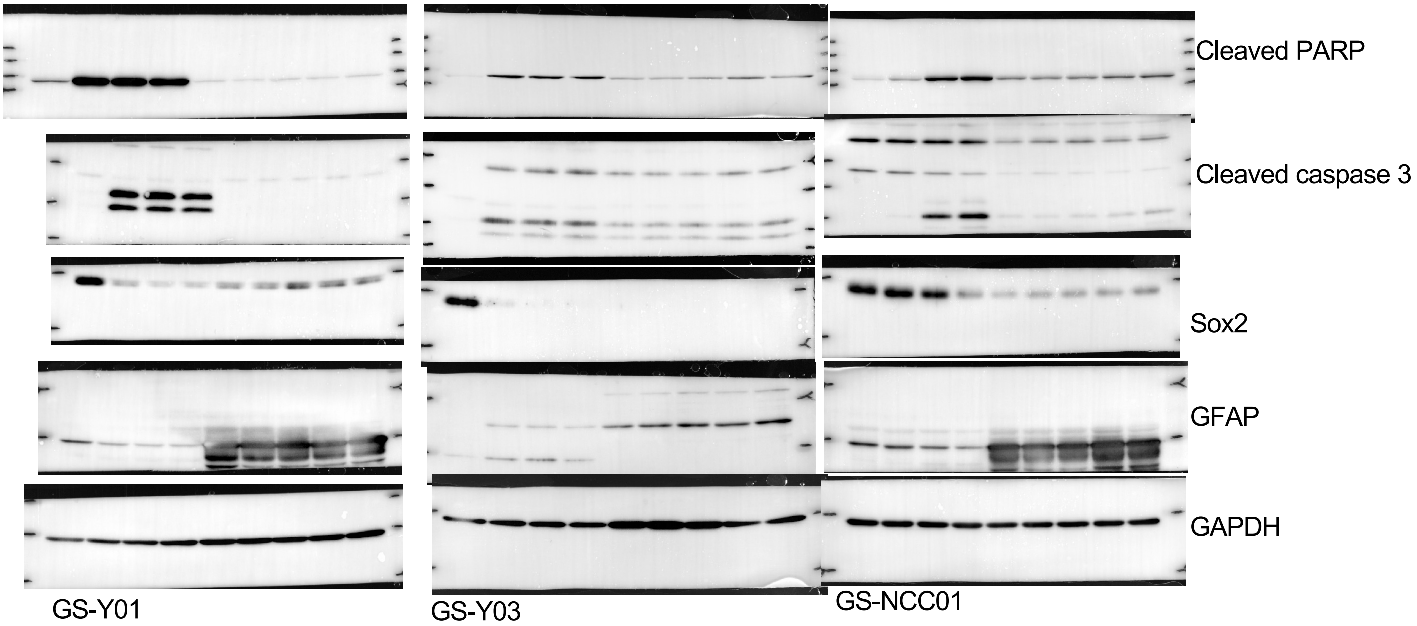

Fig3B

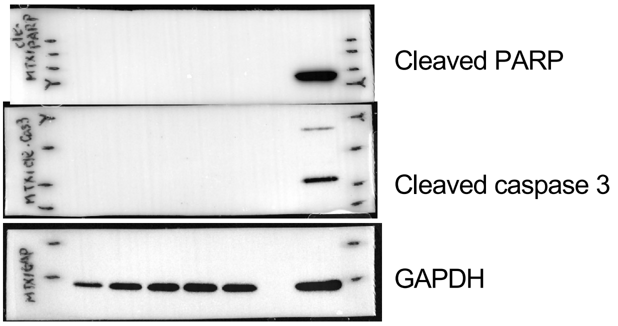

Fig3C

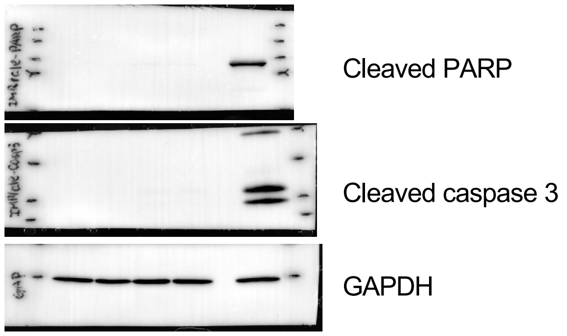

Fig4A

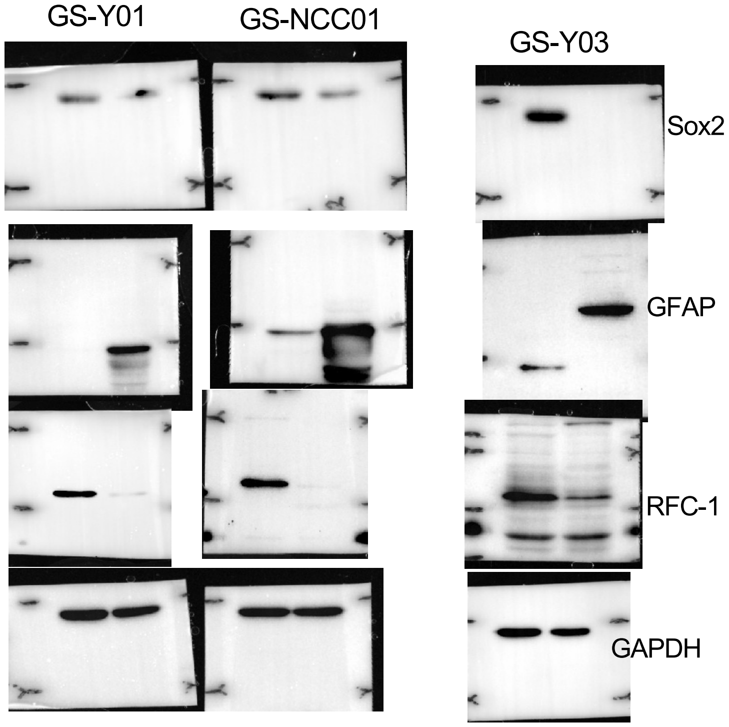

Fig4B

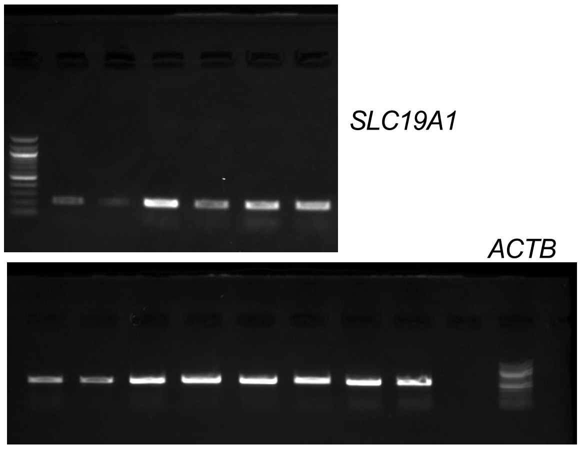

Supplemental Figure S7 (continued)

Fig5A

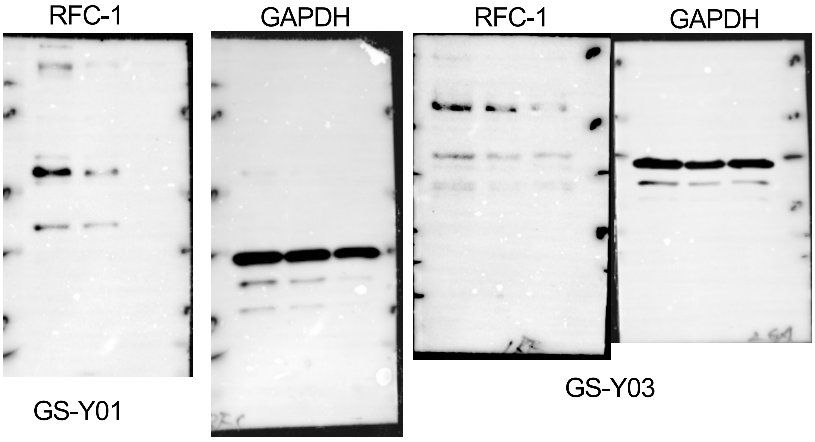

FigS1C

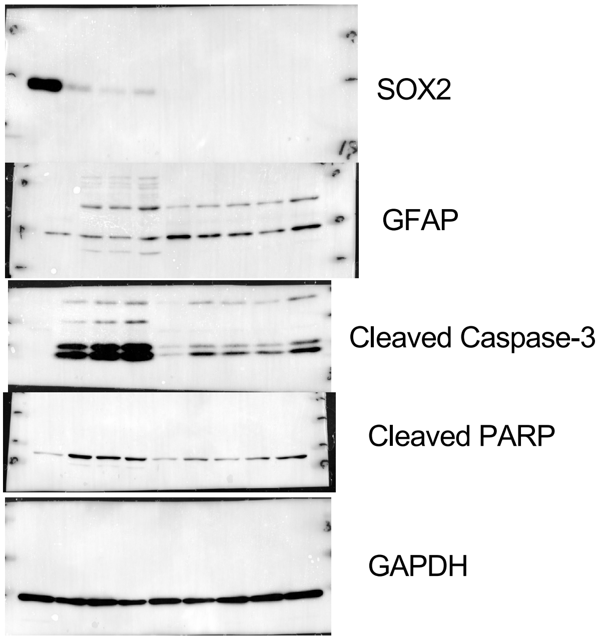

FigS2

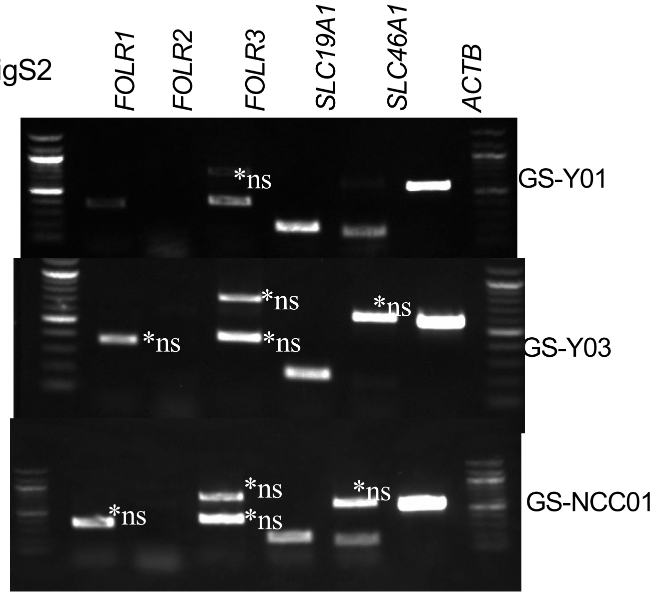

FigS5A

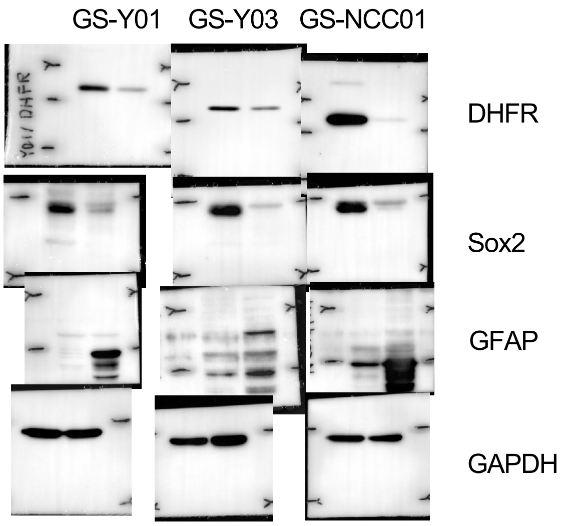

FigS6

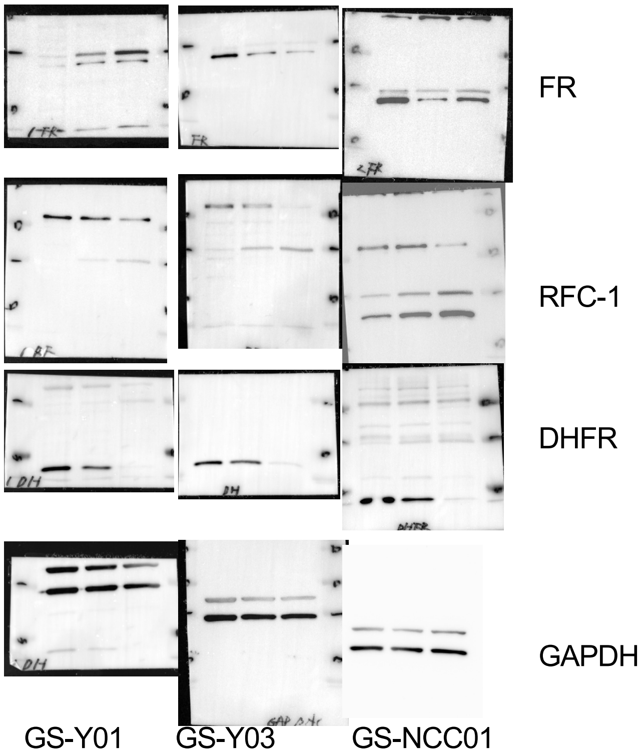

FigS5B

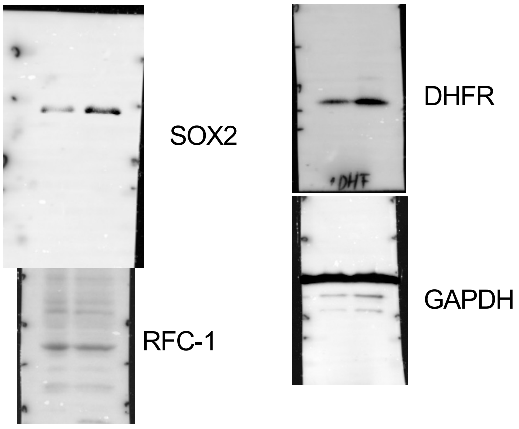

Supplemental Table S1

Sequences of primers for reverse transcription PCR analysis

Gene name	Forward	Reverse
FOLR1	CCAGCAGGTGGATCAGAGC	TTGCACAGAACAGTGGGTGT
FOLR2	AGGACAAGCTGCATGACCA	CCAGCTCTGATTCACCTGC
FOLR3	CACCTGCAAGCGCCACTTTA	CATTAATCCCTGAGGTCCAATTCC
SLC19A1	TGGTCCAAGCTGCTCATCG	GGCAAAGAACGTGTTGACCC
SLC46A1	CTTCCTGGCCAACTTTGCCTT	CCCACGTTCATGTAGAGGGTC
ACTB	CCCATGCCATCCTGCGTCTG	CGTCATACTCCTGCTTGCTG
